# Understanding the psychological nature and mechanisms of political trust

**DOI:** 10.1371/journal.pone.0215835

**Published:** 2019-05-15

**Authors:** Joseph A. Hamm, Corwin Smidt, Roger C. Mayer

**Affiliations:** 1 School of Criminal Justice, Michigan State University, East Lansing, Michigan, United States of America; 2 Environmental Science and Policy Program, Michigan State University, East Lansing, Michigan, United States of America; 3 Department of Political Science, Michigan State University, East Lansing, Michigan, United States of America; 4 Poole College of Management, North Carolina State University, Raleigh, North Carolina, United States of America; Saint Peter's University, UNITED STATES

## Abstract

Political trust is a perennially important concern and the events of the last few years have, in many ways, heightened this importance. The relevant scholarship has done much to meet this challenge but continues to struggle with definitional unclarities and an inability to provide accounts that consistently operate as expected. The current research seeks to test the potential of a classic model of trust from the organizational sciences that makes specific arguments regarding the psychological nature and mechanisms of the construct in helping to address these concerns. Using data from a national convenience sample, we provide preliminary evidence which suggests that measures and models addressing this theoretical account of psychological trust form unidimensional and reliable measures that may more precisely explain the process of political trust and outperform current measures in predicting relevant correlates. We conclude by discussing the implications and limitations of our work and, in so doing, lay a foundation for a new research agenda for political trust.

## Introduction

Political trust matters [1]. There are certainly important reasons for a society to hold a healthy level of skepticism towards its government [2], but when governments are unable to engender the trust of their constituents they open the door to a host of social costs, ranging from a lack of civic compliance with government orders (e.g., [3]) to opposition to new government programs or efforts to increase security (e.g., [4]), and may even facilitate civil conflict or separatism (e.g., [5]). It is therefore vital that research remain vigilant in understanding the nature of this trust and, to this end, social scientists have expended considerable effort. Nonetheless, and despite important advances (see [6]), political trust remains a complicated construct, often most clearly defined by measures which are themselves bogged down by continual concern over what they actually represent (see [6,7]). It is, as yet, unclear whether measures like those used in the American National Election Study (ANES), the World Values Survey, or the General Social Survey actually assess trust or whether they integrate related constructs like trustworthiness, satisfaction, or confidence.

Somewhat relatedly, research addressing political trust has struggled with identifying major drivers of the construct that consistently operate as expected. Although believed by most to be fed by “many streams” ([8] p. 12), traditional accounts of the major antecedents of political trust tend to focus heavily on performance evaluations of government. Research clearly hypothesizes that as satisfaction with various governmental outputs or processes fluctuate, so too will levels of trust in government [9]. This perspective is certainly attractive given its straightforward nature, but ultimately does not consistently explain the trends observed in actual data. Instead, scholars have had to offer a variety of alternative explanations as to why, for example, a strong trend of increasing satisfaction with the economy among the American public in the late 1980s and early 1990s failed to lead to increased trust (e.g., [10,11]).

This lack of clarity regarding the nature and sources of political trust has been a significant impediment to this important area of contemporary scholarship and, as a result, this literature provides only limited guidance for efforts to understand or predict trends in political trust. We propose that this research area could take an important step in addressing these limitations by integrating arguments from a classic model of trust from the organizational sciences [12]. We suggest that the integration of this well-accepted perspective facilitates a more precise understanding of trust in government that directly addresses what is increasingly argued to be the essence of trust across contexts, that is, the trustor's willingness to accept vulnerability [13]. We further suggest that by integrating this model’s theoretical postulations regarding the psychological conduits of trust, researchers and practitioners will be able to offer increasingly nuanced explanations of the state of political trust and, in so doing, better position themselves to accurately predict and address public reactions to various hypothetical and factual events. We then provide preliminary empirical evidence regarding the utility of this approach and discuss its potential for stimulating a new research agenda for political trust.

### MDS model of trust

In 1995, psychologists working in the organizational sciences introduced a definition and model of trust and its major drivers that has become the dominant approach to the construct. Premising on what has subsequently been argued to be a fundamental human dilemma [14], this Mayer, Davis, and Schoorman (MDS; [12]) model of trust recognizes that all human interactions carry with them the reality that the actions of the other can bring harm to the focal individual. Trust then, is the trustor’s willingness to accept that potential and is driven by propensity to trust—the trustor’s trait level predisposition to trust others generally—and trustworthiness—the trustor’s multidimensional evaluation of the trustee’s worthiness of being trusted. This model, therefore, makes a distinction that is often muddied in work addressing trust, namely that trust itself is importantly different from the things that drive it (see also [15]). This matters because although evaluations of the trustee (trustworthiness) and the personality of the trustor (propensity to trust) may lean in favor of internalizing—or not—a willingness to accept vulnerability, trust does not presuppose the existence or absence of any specific driver.

The model further proposes that there are three important dimensions of trustworthiness: ability, benevolence, and integrity. Ability addresses the perceived technical competence of the trustee within the domain of interest. It, therefore, consists of a subjective evaluation of the various skills and capabilities that may be needed for the trustee to actually accomplish what it is being trusted to do. Benevolence deals with the extent to which the trustor believes the trustee cares about and would expend effort to protect the trustor’s well-being, especially when contrasted with the trustee’s own self-interest. Finally, integrity deals with the perception that the trustee follows a set of internalized values the trustor finds acceptable. These need not necessarily be the trustor’s own values but rather a consistent set that make sense, and are acceptable, to the trustor. Thus, the MDS model selects from among a universe of potentially important evaluations of a trustee (see [16]) three especially critical evaluations that parallel arguments from the sociology of trust (e.g., competence and fiduciary responsibility; [17]) and the psychology of impression formation (e.g., warmth and competence; [18]).

There are several features of this MDS model that warrant particular emphasis here. The first is that it describes trust at a fundamental level. Instead of focusing on context-specific evaluations, it addresses underlying themes that have been argued to be consistent across contexts [13]. Thus, although the model was originally posed to describe interpersonal trust in organizational settings, it is explanatory in a variety of contexts; can be scaled to address relationships at the interpersonal, intergroup, and interorganizational levels; and even supports cross-level inference (see [19]). This general focus is further facilitated by a second major feature of the model, which is its relatively high degree of parsimony. Within MDS model scholarship, considerable effort has been expended to identify the fewest number of variables that would explain the greatest percentage of variance across the greatest number of situations. Thus, although a wide variety of trustworthiness constructs have been shown to be predictive in various contexts, research suggests that addressing ability, benevolence, and integrity is often sufficient for capturing the important variability in the construct [16]. These features, coupled with the considerable empirical support for the model, have led some to argue that this approach may be a foundational part of a cross-boundary understanding of the social science of trust [13].

### Applying the MDS model to government

Our argument for the utility of applying the MDS model to the political context lies in two central arguments: 1-that the reason that evaluations of the outputs of government matter is because of their influence on perceptions of government’s trustworthiness, and 2-that measures addressing a willingness to accept vulnerability better capture the essence of political trust itself. It is impossible to read the political trust literature and not recognize that evaluations of governmental performance play a major role in determining citizen trust. Three evaluations that have received particular attention are evaluations of the economy (e.g., [20]), whether government represents the interests of the trustor (e.g., [21]), and scandals (e.g., [22]) and, although this research does suggest important roles for these constructs, it also reveals that their influence is not consistent enough to support a direct effect [10]. Although some have argued for moderated effects like priming or polarization [11], one potential explanation for this lack of consistency may simply be that these factors are too far removed from trust to be its direct cause. The proposed integration of the MDS model directly addresses this potentiality by suggesting that between these performance factors and trust exist broadly applicable psychological conduits that mediate the effect. Specifically, this integration suggests that evaluations of government performance influence trust *because* they drive evaluations of government’s trustworthiness and, more precisely, its ability, benevolence, and integrity.

If supported, this postulation may help to explain the inconsistency in the relation between performance evaluations and trust in government. Take, for example, the recurrent discussion of the impact of the state of the economy on trust which suggests that when the economy struggles more, trust in government will be lower (e.g., [23]). Integration of the MDS model would suggest that the reason for this effect is that the struggling economy is taken by the public as evidence of a less trustworthy (e.g., less able) government which, in turn, reduces trust. Thus, although it might be expected that improvements in the economy would also improve trustworthiness assessments, the fact that they rest in a social cognition regarding the trustee means that there is good reason to believe that it would not. Application of psychological research on the relative diagnosticity of positive and negative information (e.g., [24]) would suggest that although negative information, like a worsening economy, is likely to be indicative of more negative characteristics of those perceived to have control over it, positive information does not necessarily mean that those characteristics have improved.

This is an important departure from more traditional notions of political trust, which often assume a direct link to performance evaluations, but it also builds upon more nuanced arguments that integrate moderators of this direct effect. In particular, many have argued that consistency with previous attitudes (e.g., [25]) or salient identities (e.g., [26]) may trigger motivational processes such that, when the attitudes and identities of those in power match their own, individuals are more likely to be unpersuaded by negative performance and more likely to be persuaded by positive performance. Researchers have also argued that attentional issues may break the relation between performance evaluations and trust such that individuals may be focused on one element of government when thinking about performance, and another when thinking about trust [25]. Although important, this additional nuance still neglects the likely role played by attributions arising from performance evaluations. Thus, although we know that individuals are more likely to attribute positive performance to political parties with whom they share salient values [27], there is more to be said about why this would impact trust. Integration of the MDS model formally addresses this by hypothesizing that this effect is the result of trustworthiness attributions such that when trustors and people in power share ideologies, positive performance signals trustworthiness more readily than when these ideologies do not match.

The second contribution of integrating the MDS model lies in its implications for the conceptualization and operationalization of the construct itself. Research addressing political trust in the US typically finds its core in four items from the American National Election Study (ANES) and, despite a lively debate as to what this index or its components actually measure, this scholarship has largely defined the construct by it (e.g., [22,28,29]). This consistency in measurement has served the field well by permitting significant national and even international longitudinal analyses over the last fifty years, but it has also prevented the field from taking advantage of the noteworthy conceptual advances in the wider social science of trust, especially during the last two decades. This research generally accepts that, in order for trust to be relevant, it must refer to a relationship in which the trustee has the *agency* (see [30]) to make intentional decisions that impact the harm that may befall the trustor [31]. It is important to note that there is, as yet, no consensus as to the extent or type of harm necessary, but research has considered negative outcomes that run from direct personal harm to more amorphous violations of notions of what the trustee should be (e.g., [32]). Applied to political trust, this would mean that the public’s trust is rooted in an awareness that the deliberate actions of government can intentionally or unintentionally impact the probability or severity of potential harm. As in other contexts, the range of these potential harms is quite broad and includes concerns like outright persecution, intended or unintended impediments to the individual’s access to the means necessary for the standard of living to which they feel entitled, or intrusions into otherwise unimportant areas of life that the individual simply feels are not within the purview of government. Thus, from the perspective of the MDS model, trust is premised on this vulnerability, but focuses specifically on the individual’s willingness to accept it such that a trusting individual is more or less consciously willing to accept the reality that the agentic actions of the other could impact the potential for or severity of harm [31,33]. Measures applying this conceptualization therefore typically focus on comfort with, a willingness to increase, or a lack of motivation to decrease that dependency (e.g., [32,34]).

Evaluation of the ANES index from this MDS model perspective both helps explain the persistent utility of the measure and highlights areas in which it could be improved through reconceptualization or reinterpretation. The index includes four items that ask the participant to indicate: 1-how often the “government in Washington” can be trusted to do what is right, 2-the extent to which it is run by special interests versus the people, 3-whether money paid in taxes is wasted, and 4-the proportion of people running government that are “crooked.” It is therefore readily apparent that, in place of the conceptualization of trust proposed within the MDS model, these items focus on evaluations of government itself. As a result, despite being likely to correlate with direct measures of the internalized state that we argue to be trust, the ANES index seems to more squarely address trustworthiness. The first item—variants of which Gallup and recent comprehensive studies [11] rely on to measure trust—asks the participant to consider their own internalized beliefs about how often government will “do what’s right”. This, in MDS terms, refers most directly to an evaluation of government’s integrity but may also infer some level of ability to actually do the right thing or benevolence in identifying the “correct” right thing. The second item appears to tap into benevolence and, depending on how the trustor feels about the moral codes of “special interest groups”, may also address integrity. The third item concerns waste, which likely taps into ability-related inefficiency but may also address integrity-related use of government funds for reasons the trustor deems inappropriate. Finally, the fourth item seems to most squarely assesses the trustor’s perception of government’s integrity.

The utility of the ANES index may therefore lie in the reality that, although not specifically intending to do so, the measure addresses constructs that are important within the MDS model, but the extent to which the individual items precisely target any of its hypothesized dimensions is unclear. Most of the items could be read as implicating evaluations of whether government has the ability to do what it is trusted to do, the benevolence to care for the trustor and people like them, *and* the integrity to do the right thing. Thus, although the use of the index likely provides values that correlate well with MDS notions of trust, ability, benevolence, and integrity, combining these distinct constructs into a single measure dilutes the clarity of any of them. This imprecision precludes a nuanced diagnosis of the relative levels or relations among these specific trust and trustworthiness constructs, but it may also reduce the measure’s responsiveness to change in any one causal factor. For example, in the face of an improving economy, it might be expected that perceptions of government’s ability will rise but that this improvement would not affect its perceived benevolence or integrity. In such a case, the effect of improvements in the economy on trust might be diluted by the inclusion of the latter two factors within the ANES such that even moderate increases in one dimension may be “averaged out” of the index score. Thus, a major part of the proposed value of the integration of the MDS approach into the scholarship on political trust is that it would enable scholars to reconceptualize a longstanding survey measure of political trust within a more delineated psychological framework.

### The current research

**Fig 1:**
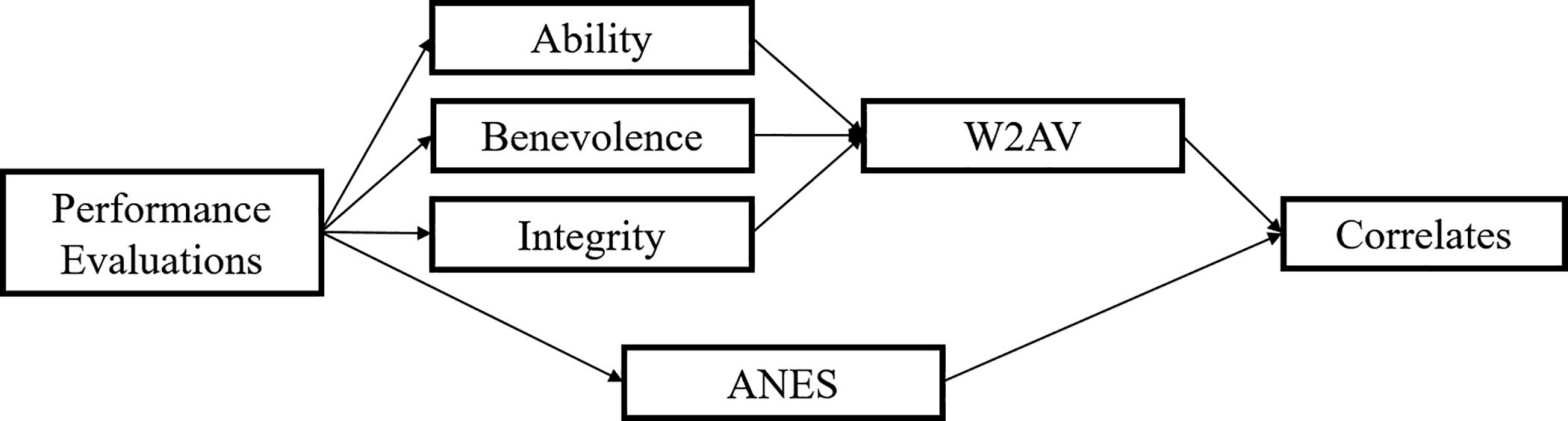
Study hypotheses. Unidirectional arrows indicate regression paths.

The central purpose of the present work is to provide preliminary evidence regarding the utility of integrating the MDS model into political trust scholarship. We suggest that this model provides more nuanced conceptualizations of trust, its related constructs, and of the psychological mechanisms that connect them, and that this increased precision will lead to greater empirical utility for measures that apply it. More specifically, we hypothesize 1-that MDS-grounded measures of political trust and trustworthiness will form reliable and unidimensional constructs; 2-that trust, measured using the MDS notion of a willingness to accept vulnerability (*W2AV*), will be more strongly related to relevant correlate measures than trust measured by the ANES index; and 3-that trustworthiness assessments of government’s ability, benevolence, and integrity will mediate the relation between performance evaluations of government and *W2AV* (see Fig 1). To test these hypotheses, the current research uses cross-sectional data collected from a national convenience sample. Thus we present this work, not as a definitive test of the proposed model, but as an empirical foundation upon which subsequent work—and especially longitudinal work with more representative samples—can build.

## Materials and method

A national convenience sample of participants (*n* = 503) were recruited using Mturk to complete an online survey about their perceptions of the federal government during the fall of 2015. To address potential attention and motivation issues, participation was restricted to Master Workers who had approval ratings of 95% or greater. The selection criteria for these individuals is proprietary but is intended to identify high-performers and is therefore associated with a higher required wage. Although not without concerns in social science research (e.g., [35]), work addressing these samples is generally supportive of their use and suggests that they are both more representative (e.g., [36]) and no more likely to elicit problematic responding (e.g., [37]) than more traditional approaches. Thus, although research does suggest important differences in demographic and other characteristics (e.g., comfort using technology), the current sample represents a sufficient and—given the trade-off between cost and utility—potentially even ideal basis for such preliminary work [36].

The sample self-reported an average age of 38 years, was roughly evenly divided on sex (49.9% female), and was primarily White (77.5%). About half of the sample had not completed a bachelor’s degree (55.3%) and 83% percent of the sample reported an annual income of less than $60,000 per year. Politically, the sample self-reported as more Democrat (39.9%) than Republican (17.3%; 42.8% Independent or leaning) and was more liberal on social (66.2%; 20.8% conservative), economic (47.4%; 35.1% conservative), and general ideology (53%; 22.9% conservative).

### Survey

Upon providing informed consent, participants were randomly assigned to one of three priming conditions in which they read a brief factual statement regarding the Office of the President of the United States, the United States Congress, or the Supreme Court of the United States (see S1 File). These sections were assembled from a variety of open sources and were designed to simply present unequivocal factual information. Participants were then asked to write a brief paragraph addressing their thoughts about the assigned institution. This manipulation was intended to permit evaluation of concerns from the literature regarding the effect of priming on political trust. This research suggests that when individuals are primed to think about the aspects of government that they feel more or less positively about, political trust will similarly vary (e.g., [9,25]). This manipulation was included to permit evaluation of the relative sensitivity of the MDS constructs for each priming condition but, surprisingly, omnibus bivariate analyses associating this manipulation with our constructs of interest revealed no significant differences by condition. This suggests either that none of the constructs were impacted by priming or that our manipulation failed. We therefore collapsed across conditions for all following analyses.

The next section assessed participants’ perceptions of government and their willingness to cooperate or comply via a variety of correlate measures (see S1 Table for measure wording, univariates, and response scales). The block started with three performance evaluations that asked whether the participant believed that government had generally helped or hurt the economy, represented their interests, and whether government scandals have been a big deal over the last few years. Participants next completed the standard ANES trust index and our new trustworthiness measure which was comprised of three, three-item scales addressing ability, benevolence, and integrity. Because of their relative semantic and conceptual similarity, the trustworthiness and ANES items were presented together in counterbalanced order. Participants then completed our three-item measure of trust conceptualized as a willingness to accept vulnerability (*W2AV*).

Participants next completed a series of correlate measures which were selected to address a variety of contexts in which political trust has been shown to be important. We therefore included a feelings thermometer, a question assessing whether the participant felt they pay too much or too little in taxes, the participant’s willingness to encourage cross-party cooperation, their support of federal government monitoring of Americans, and their willingness to comply with an evacuation order or a recommended vaccination. Thus, our correlates sought to address three concepts that are common in discussions of political trust; namely, global attitudes (i.e., the feelings thermometer; [11,23]), a willingness to personally engage in potentially risky behaviors that were recommended by government (i.e., evacuation and vaccination; [3,38,39]), and a willingness to encourage government to engage in behaviors that involve some level of potential risk to the public (i.e., cross-party cooperation and monitoring of Americans; [4,40]).

The final survey section included demographics and assigned a completion code. The survey took approximately 10 minutes to complete and participants were compensated $1.70 for their time. All procedures were approved by the Human Research Protection Program at Michigan State University (x15-502e). Participants provided informed consent as part of the online survey.

## Results

### Hypothesis one

To address Hypothesis One (that the proposed measures form reliable and unidimensional latent constructs in this context) we conducted a confirmatory factor analysis (CFA) of our new measures of *Ability*, *Benevolence*, *Integrity*, and *W2AV* in M*plus* version 6 using the Maximum Likelihood-Robust estimator. The model fit well to the data (*χ*^*2*^ (48) = 71.01, *p* = .02; RMSEA = .03, *p* = .99; CFI = .99; TLI = .99; SRMR = .02) and revealed all items to have significant loadings on their hypothesized factors (see Table 1). This good global fit was corroborated by evaluation of the modification indices which suggested very little local misfit. In fact, the only noteworthy recommended model change suggested that the second *W2AV* item was less related to the scale’s other items than they were to each other. Given the good reliability of the latent factor and the item’s strong loading, however, we did not incorporate this modification.

**Table 1:** Confirmatory factor analysis results and item univariates.

Construct	Item	Latent Var. Rel. (*ω*)	Std. Load. (*λ*)	Mean	SD
*Ability*	The federal government is generally competent.	.84	.87***	2.92	0.83
	The federal government is capable of performing its job.		.85***	3.08	0.84
	The federal government has the knowledge necessary to do the work that needs to be done.		.67***	3.37	0.89
*Benevolence*	The federal government cares about people like me.	.94	.92***	2.44	0.83
	The federal government is concerned about the welfare of people in situations like mine.		.92***	2.49	0.83
	The federal government looks out for what is important to people similar to me.		.91***	2.49	0.82
*Integrity*	The federal government sticks to its word.	.86	.85***	2.64	0.80
	The federal government adheres to a strong moral code.		.82***	2.40	0.88
	The words and actions of the federal government are consistent.		.78***	2.60	0.85
*W2AV*	I am open to letting the federal government make more decisions about issues that are important to me.	.90	.86***	2.44	0.92
	I am comfortable with the federal government’s control over my future.		.89***	2.27	0.92
	I am willing to let the federal government resolve problems that are critical to me, even though I cannot monitor all of its actions.		.84***	2.58	0.97

***p < .001

The CFA also suggested strong correlations among the latent constructs (see Table 2). Because of their ability to partial out statistical noise, latent analyses often increase correlations between constructs but especially large coefficients may be indicative of an over-parameterized model (one in which the model makes more distinctions than participants did). As a result, researchers are encouraged to test alternative model specifications that may better represent the data [41]. We, therefore, tested both a single factor solution (in which the items that indicated the three trustworthiness factors were entered as indicators of a single latent factor) and a higher-order factor solution (in which a higher-order latent factor was entered “above” the three latent trustworthiness factors). Both models fit numerically worse than the original, correlated factors model, but the decrease in scaled log-likelihood per degree of freedom for the higher-order factor model (-2ΔLL/df = 2.67, *p* = .06) was not statistically significant and was much smaller than the single factor model (-2ΔLL/df = 45.02, *p* < .001). We therefore accepted the higher-order factor model as the most parsimonious and statistically defensible representation of our data.

**Table 2:** Latent and ANES variable correlations.

Construct	*Ability*	*Benev*.	*Integ*.	*ANES* *Index*	*ANES* *1*	*ANES* *2*	*ANES* *3*	*ANES* *4*
*Ability*	-	-	-	.63***	.47***	.27***	.16**	.16**
*Benevolence*	.75***	-	-	.70***	.50***	.11**	.24***	.11*
*Integrity*	.87***	.86***	-	.72***	.48***	.21***	.23***	.17**
*W2AV*	.72***	.68***	.75***	.63***	.44***	.11*	.29***	.10^+^

^*+*^*p* < .10

*p < .05

**p < .01

***p < .001

Before testing the remaining hypotheses, we also tested additional models to determine the relations among the latent *W2AV* and *Trustworthiness* factors and the ANES measure modeled first as its composite index and second as individual items (see the right panels of Table 2). The ANES index was more correlated with the four latent constructs than were the items but was largely indiscriminant such that it correlated approximately as strongly with *Ability*, *Benevolence*, *Integrity*, and *W2AV*. The ANES items were similarly indiscriminant but the question asking whether government can be trusted to do what’s right was generally associated with larger correlations with all four MDS constructs.

### Hypothesis two

We tested Hypothesis Two (that W2AV would be more strongly related to the correlate measures than would the ANES index) using latent, multivariate models, again in M*plus*. In preparation for these structural analyses, we first estimated a saturated measurement model in which all correlations among our variables of interest were freely estimated. The model fit well to the data (*χ*^*2*^ (150) = 234.09, *p* < .001; RMSEA = .03, *p* > .99; CFI = .99; TLI = .98; SRMR = .03) and suggested that the strongest relations were among *W2AV*, *ANES*, and *Trustworthiness*, each of which had significant relations with all of the other variables in the model (see Table 3). *Eval*. *Economy* and *Eval*. *Interests* were also significantly associated with all of the correlate measures but it is worthy of note that *Eval*. *Scandals* was only significantly correlated with the *Tax Item*, the *Feelings Thermometer*, and *Vaccination*. Indeed, *Eval*. *Scandal*s was generally less correlated with all of the other variables in the model than were the other performance evaluations. Evaluation of its mean and variability, however, suggested that participants neither rated this variable particularly negatively nor that its variance was notably smaller than the other evaluative constructs (see S1 Table). Thus, it seems that this restriction in effect is less likely to be a measurement artifact and more likely to be a real conceptual distinction—we will return to this point in the discussion.

**Table 3:** Saturated model variable correlations heat map.

Construct	1	2	3	4	5	6	7	8	9	10	11
1. *W2AV*											
2. *ANES*	.63***										
3. *Trustwor*. *(HOF)*	.79***	.76***									
4. *Eval*. *Economy*	.51***	.48***	.56***								
5. *Eval*. *Interests*	.62***	.58***	.72***	.66***							
6. *Eval*. *Scandals*	.32***	.34***	.30***	.41***	.38***						
7. *Tax Item*	.46***	.30***	.38***	.34***	.35***	.30***					
8. *Feelings Therm*.	.72***	.69***	.83***	.59***	.64***	.33***	.37***				
9. *Cross-Party Coop*.	.27***	.14***	.28***	.16**	.16**	.05	.13*	.23***			
10. *Monitoring*	.30***	.28***	.31***	.12**	.22***	.05	.04	.30***	.18***		
11. *Evacuation*	.29***	.12**	.28***	.16**	.21***	.06	.13**	.25***	.24***	.14**	
12. *Vaccination*	.42***	.31***	.34***	.30***	.30***	.20***	.27***	.32***	.14**	.16**	.38***

Values without superscript are not statistically significant. HOF = Higher-Order Factor.

*p < .05

**p < .01

***p < .001

**Fig 2:**
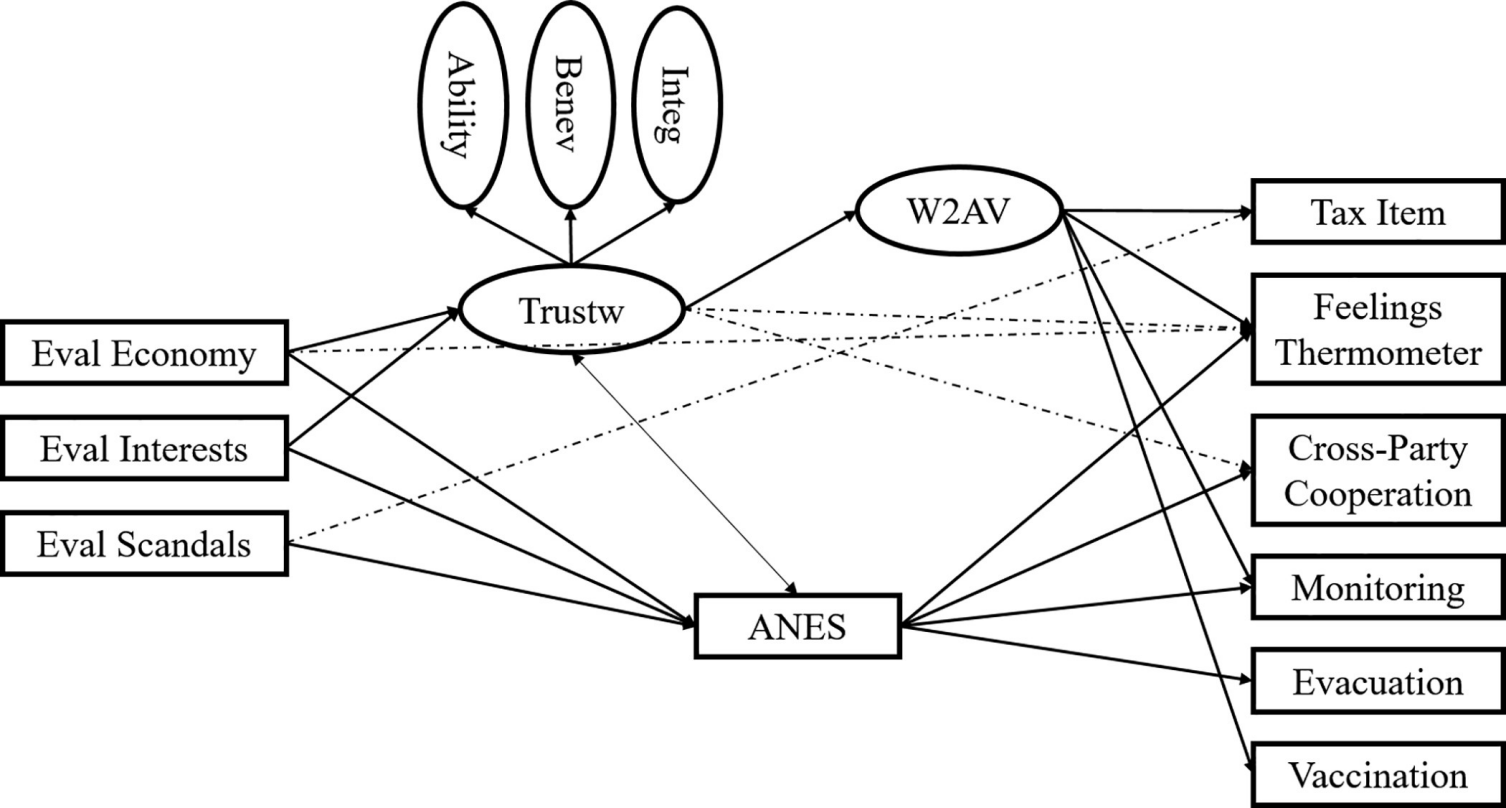
Significant relationships within the saturated structural equation model. Solid lines indicated significant hypothesized effects. Hashed lines indicate significant non-hypothesized effects. Demographic variables were included in the model (see Table 4) but are suppressed for readability.

We then estimated a structural equation model (SEM) in which each of the correlates were regressed onto *W2AV*, *ANES*, *Trustworthiness*, all three performance evaluations, and demographics (see Table 4). To address all possible relations, additional paths were estimated in which *W2AV* was regressed onto the performance evaluations and demographics in addition to *Trustworthiness*. *Trustworthiness* was also directly regressed on the evaluations and demographics. Finally, to address the relation between *Trustworthiness* and *ANES* suggested by their strong correlation, an additional path was included linking these constructs. This model was therefore saturated in that regression coefficients were estimated testing the effects of all possible predictors of each construct (i.e., all criteria were predicted by all variables that appear to their left in Fig 1). The model again fit well to the data (*χ*^*2*^ (191) = 289.71, *p* < .001; RMSEA = .03, *p* = .99; CFI = .98; TLI = .98; SRMR = .02) and revealed most of the hypothesized pathways (see Fig 2). Regarding the correlates, *W2AV* generally had the strongest independent relation but this was not always true—both *Feelings Thermometer* and *Cross-Party Cooperation* were better predicted by *Trustworthiness* directly (see Table 4). Additionally, although the regression coefficient for the effect of *Trustworthiness* on *Evacuation* was numerically largest, *ANES* had the only significant predictive relation but it is important to note that this may have simply been a result of the variable’s relatively larger range (see S1 Table).

**Table 4:** Saturated structural equation model results.

Criterion	Predictor (*β*)										*R*^2^
	*W2AV*	*ANES*	*Trustwor*. *(HOF)*	*Eval*. *Economy*	*Eval*. *Interest*	*Eval*. *Scandals*	*Educ*.	*Inc*.	*Part*.	*Part*. *Strength*	
*Taxes*	.33***	-.02	.02	.10	.01	.12*	.02	-.03	-.10^+^	.01	.25***
*Feelings Therm*.	.13*	.10*	.56***	.17***	.03	.03	.04	-.01	.02	.04	.73***
*Cross-Party Coop*.	.11	-.17*	.37**	.05	-.13^+^	-.05	.02	.14**	-.08	.01	.12***
*Monitoring*	.23*	.11	.09	-.10	.02	-.03	-.06	.15**	.11*	.01	.16***
*Evacuation*	.19^+^	-.18*	.25^+^	.001	-.01	-.03	-.08	.06	-.06	.09	.13***
*Vaccination*	.32***	.08	-.04	.11^+^	-.02	.02	.04	.01	-.12*	-.04	.20***
*W2AV*	-	-	.71***	.03	.04	.04	.04	.01	-.14***	.002	.66***
*ANES*	-	-	*r* = .61***	.16**	.46***	12**	.04	-.01	.11**	.01	.39***
*Trustwor*. *(HOF)*	-	-	-	.16**	.61***	.02	.02	.02	.06	.12**	.56***

Values without superscript are not statistically significant. HOF = Higher-Order Factor.

^*+*^*p* < .10

*p < .05

**p < .01

***p < .001

In order to address potential concerns about the greater potential for error in sophisticated techniques like latent variable modeling, we also tested this central argument of Hypothesis Two research by simply evaluating the significance of the difference between dependent zero-order correlations of observed variable item averages the MDS construct scales and ANES index with the correlates (see Table 5). Four of the six were significantly more strongly correlated with the W2AV measure, and none were significantly more strongly correlated with ANES.

**Table 5:** Significance of difference in zero-order correlations.

Correlates	*Trust*	*ANES*	*Z-*Score
*Taxes*	.43	.30	3.44***
*Feelings Thermometer*	.68	.69	0.44
*Cross-Party Cooperation*	.25	.14	2.89**
*Monitoring*	.28	.28	0.03
*Evacuation*	.27	.12	3.80***
*Vaccination*	.39	.31	2.29*

*Z*-scores without superscript are not statistically significant

*p < .05

**p < .01

***p < .001

### Hypothesis three

Hypothesis Three (that *Ability*, *Benevolence*, and *Integrity* would mediate the effects of the performance evaluations on *W2AV* and its correlates) was also addressed in the SEM. Recall that in the measurement model, *Eval*. *Economy* and *Eval*. *Interests* were correlated with both trust measures and all of the correlates, and that *Eval*. *Scandals* was significantly related to the both trust measures, the *Tax Item*, *Feelings Thermometer*, and *Vaccination*. In the structural model, however, most of these direct effects were non-significant, suggesting that they were largely mediated as hypothesized (see Table 4). Indeed, the only direct effects of the performance evaluations that remained significant were the regressions of the *Feelings Thermometer* onto *Eval*. *Economy* and of the *Tax Item* onto *Eval*. *Scandals* (see hashed lines in Fig 2). Although each of these effects are directly contrary to the hypothesis, the latter (*Eval*. *Scandals*→*Tax Item*) lends credence to the general finding throughout this study that evaluations of government scandals are importantly different from the other constructs in the model. In addition to these unexpected paths, our saturated model also revealed two significant direct effects of *Trustworthiness* that also appear to be unmediated (see the hashed lines in Fig 2). Although not a major focus of the current research, the MDS model suggests that the relation between trustworthiness and relevant outcomes (here represented by the correlate measures) is fully mediated by a willingness to accept vulnerability. This postulation held for most of the correlate variables, but significant direct effects were identified for *Trustworthiness* on *Feelings Thermometer* and *Cross-Party Cooperation*, independent of *W2AV*.

To directly test the mediations implied above, we evaluated the statistical significance of all possible indirect effects (see Table 6). As hypothesized, *Trustworthiness* mediated significant indirect effects on *W2AV* for *Eval*. *Economy* and *Eval*. *Interests* but, contrary to our hypotheses, this was not true for *Eval*. *Scandals*. *W2AV* then significantly or marginally mediated indirect effects of *Trustworthiness* on all of the correlate measures except *Cross-Party Cooperation*. Conversely, although *ANES* significantly or marginally mediated indirect effects of *Eval*. *Scandals*, *Eval*. *Interests* and *Eval*. *Economy* on four of the correlates, it did not mediate the impacts on *Tax Item*, *Monitoring*, or *Vaccination*.

**Table 6:** Summary of indirect effects.

IV	M	DV	Standardized Ind. Effect
*Eval*. *Economy*	*Trustwor*. *(HOF)*	*Trust*	.12**
*Eval*. *Interests*			.43***
*Eval*. *Scandals*			.01
*Trustworthiness* *(HOF)*	*Trust*	*Tax Item*	.23**
		*Feelings Therm*.	.09*
		*Cross-Party Coop*.	.08
		*Monitoring*	.16*
		*Evacuation*	.14^+^
		*Vaccination*	.22**
*Eval*. *Economy*	*ANES*	*Tax Item*	-.003
*Eval*. *Interests*			-.009
*Eval*. *Scandals*			-.002
*Eval*. *Economy*		*Feelings Therm*.	.02^+^
*Eval*. *Interests*			.05*
*Eval*. *Scandals*			.01^+^
*Eval*. *Economy*		*Cross-Party Coop*.	-.03^+^
*Eval*. *Interests*			-.08*
*Eval*. *Scandals*			-.02^+^
*Eval*. *Economy*		*Monitoring*	.02
*Eval*. *Interests*			.05
*Eval*. *Scandals*			.01
*Eval*. *Economy*		*Evacuation*	-.03^+^
*Eval*. *Interests*			-.08*
*Eval*. *Scandals*			-.02^+^
*Eval*. *Economy*		*Vaccination*	.01
*Eval*. *Interests*			.04
*Eval*. *Scandals*			.01

Values without superscript are not statistically significant. HOF = Higher-Order Factor.

^*+*^*p* < .10

*p < .05

**p < .01

***p < .001

Finally, to determine the importance of all four non-hypothesized paths for model fit, we next estimated a series of alternative models in which one of the non-hypothesized paths was set to zero. If these direct effects could thus be “turned off” without significantly impacting model fit, the effect could be argued to be completely accounted for by the remaining paths within the model. If, however, the model changes resulted in a significant drop in fit, it would suggest the existence of a meaningful direct effect such that any mediation could not be said to be complete. As reported in Table 7, all four model changes resulted in a statistically significant reduction in model fit as compared to the complete model. It is worthy of note, however, that the decrement in model fit was much larger for the two effects on *Feelings Thermometer* with the largest change in scaled log likelihood accompanying the removal of its direct relation with *Trustworthiness* (-2LLΔ/df = 753.53, *p* < .001).

**Table 7:** Path removal model comparisons.

Model Change	Comparison to Base Model
*Eval*. *Economy→Feelings Therm*.@0	-2LLΔ (1) = 44.84***
*Eval*. *Scandals→Tax Item*@0	-2LLΔ (1) = 6.19*
*Trustwor*.*→Cross-party Coop*.@0	-2LLΔ (1) = 7.70**
*Trustwor*.*→Feelings Therm*.@0	-2LLΔ (1) = 753.53***

*p < .05

**p < .01

***p < .001

## Discussion

The research reported here sought to lay preliminary empirical groundwork for a new line of scholarship on political trust that would advance the literature by integrating arguments from a model of trust from the organizational sciences that has been well-supported in a variety of contexts. This model argues that trust is a willingness to accept vulnerability to the agentic actions of the trustee and hypothesizes that this willingness is driven by an evaluation of the trustee’s worthiness of that trust. When integrated with traditional approaches to political trust, this suggests that the effects of the antecedents that are usually discussed in this literature may actually be mediated by attributions of government’s trustworthiness, which can be meaningfully captured by assessing its perceived ability, benevolence, and integrity. Three hypotheses flow directly from this integration of the MDS model and preliminary, cross-sectional support for each was provided here.

### Hypothesis one

The first hypothesis suggested that measures of trust (*W2AV*) and trustworthiness (*Ability*, *Benevolence*, and *Integrity*) that follow the MDS model conceptualizations would form reliable and unidimensional latent constructs in this context, and was tested in a CFA. As hypothesized, the model fit well to the data overall and an evaluation of local misfit suggested that no model modifications were necessary. It is, however, important to note that the correlations among the three latent trustworthiness constructs were high. We therefore tested an alternative model in which the nine trustworthiness items were included as indicators of a single factor, and one in which the *Ability*, *Benevolence*, and *Integrity* latent constructs were entered as indicators of a higher-order *Trustworthiness* factor. Although both model changes resulted in numerical decrements in model fit, the decrease associated with the higher-order factor model was notably smaller and only marginally significant. We therefore accepted this model configuration as the best representation of our data. Conceptually this suggests that the MDS model can be effectively leveraged to create psychometrically sound measures of trust in government, but the high correlations among the constructs do highlight the possibility that, despite being separate evaluations that may have varying influences in longitudinal or experimental contexts, these may yet represent “a (conceptual) distinction without a (practical) difference” in cross-sectional survey data ([32] p. 1205).

Given that the distinction between trust and trustworthiness is a centerpiece of the MDS model, it is also worthy of note that the relations between trustworthiness and both measures of trust were also relatively high. Within MDS scholarship, trust refers to the trustor’s internalized state, while trustworthiness addresses perceptions of the characteristics of the trustee that facilitate it. This matters because it is possible for an individual to find a trustee to be trustworthy and still not trust them or, potentially more interestingly, to find them untrustworthy and yet trust. The reality of the situation, however, is that this is unlikely to happen in practice. Whether because trust rests on a calculated evaluation of trustworthiness [33] or because individuals simply struggle to hold conflicting evaluations of the same institution [42], the correspondence between trust and trustworthiness is often high, especially in cross-sectional data (see [16]). Nonetheless, their statistically defensible conceptual distinction allows researchers to consider the constructs independently and, in so doing, better understand situations in which they are less related (see [15]). Indeed, our results lend credence to this by showing that trustworthiness and the measures of trust are inconsistent in their independent prediction of the correlates. Instead, trust, measured as a willingness to accept vulnerability, was typically—but not always—the best predictor. This finding, coupled with the good global and local fit of the models that included trust and trustworthiness as separate latent factors, defends our decision to model them as distinct but stops short of demanding that all future research do the same.

Our Hypothesis One results provide an important basis for future research by suggesting that the measures proposed here are effective operationalizations of the MDS approach in the political context. Thus, the current work provides a foundation for future efforts by presenting potential measures but builds upon this contribution by providing evidence of especially strong relations among the constructs. Thus, cross-sectional research that was limited in space could use the current work to support a decision to include fewer items, for example, by including one *W2AV* item and one from each of the trustworthiness measures. Given their potential for greater nuance, longitudinal efforts would likely be best served by including the complete measures, but could rely on the current analyses to anticipate effect sizes.

### Hypothesis two

The second hypothesis—that trust measured as a willingness to accept vulnerability would more strongly predict a variety of important correlate measures than trust measured by the ANES index—was also generally supported in our data. The bivariate relations among latent constructs reported in Table 3 suggested that although both measures of trust were correlated with all of the other constructs in our research, trust measured as a willingness to accept vulnerability had a numerically higher average correlation with the performance evaluation constructs (*r*_avg_ = .59) and correlates (*r*_avg_ = .39) than did trust as measured by ANES (*r*_avg_ = .47; *r*_avg_ = .31). This stronger relation was also generally supported in the structural model where the latent *W2AV* factor had the strongest independent effects on value for taxes, acceptance of monitoring, and willingness to comply with vaccination recommendations. Contrary to the hypothesis, *ANES* was the only significant predictor of a willingness to comply with an evacuation order, but it is important to note that the effects of both *W2AV* and *Trustworthiness* were associated with numerically larger regression coefficients suggesting that the significance of *ANES’* effect may have been rooted in its relatively larger range.

Our results also suggest that, *Trustworthiness* was the strongest predictor of both the feelings thermometer and a desire for cross-party cooperation. This finding directly contravenes our hypothesis and a core argument within the MDS model but may make some sense in light of the conceptual distinction between the constructs. As noted in the introduction, a core argument of the MDS model is the postulation that a willingness to accept vulnerability mediates of the effect of trustworthiness on risk taking in the relationship. The lack of a significant direct effect of trustworthiness on most of the correlates is consistent with this postulation but its significant direct effects on both the *Feelings Thermometer* and *Cross-Party Cooperation* seem to directly contradict this unless these correlates do not represent risk taking. Thus, although some have extended this mediation to all instances of cooperation and compliance, it remains possible that trust only mediates the effect of trustworthiness when the dependent variable involves salient risk taking. Paying taxes, accepting monitoring, evacuating, and being vaccinated all involve some level of potential for harm, if only in hypothetically deciding to expend the time and money needed to be vaccinated. The *Feelings Thermometer* however, may not directly infer any level of risk taking insofar as this is simply an evaluation of the trustee. Thus, it may be better conceptualized as an alternative evaluation of government (akin to trustworthiness). The significant direct effect of *Trustworthiness* on *Cross-Party Cooperation* however is somewhat more curious. This variable is certainly something more than a simple evaluation of government and does seem to involve some level of risk taking in that asking one’s political party to work with others implies empowering your party to make concessions. Our data however, seem to suggest that trustworthiness not only maintains a significant direct effect on this correlate, but that it actually displaces the effect of trust measured as a willingness to accept vulnerability. Future research is needed to more fully understand this.

These results contribute to future research by providing preliminary evidence that the MDS model constructs may outperform the ANES index. As noted in the introduction, data using the ANES index have been collected for decades and currently underpin a majority of the scholarship on the construct, but this prominence obscures potential for improvement. Our results regarding *W2AV* show demonstrable, and in most cases, statistically significant improvement. It is important to note that this improvement was somewhat modest in the current sample but these relations may be somewhat conservative given the use of cross-sectional data (which likely inflates common-method variance) and online participation (which may introduce noise from less-than-careful responses). Conversely, it is also important to note the statistical advantages of the MDS model constructs, most notably the use of nine items (distributed across three latent factors) as indicators of the higher-order trustworthiness factor. Thus, although future work is certainly needed, the current, preliminary analyses provide some level of confidence that the empirical utility of the MDS model constructs is at least as strong as that of the ANES.

To be clear, these results should not be read as a reason for discontinuing the use of the ANES index altogether. Indeed, the historical use of the index alone provides a sufficient rationale for its persistence. Instead, these analyses suggest that interpreting the ANES index from the perspective of the MDS model and supplementing its measurement with MDS model constructs may provide a much needed basis for future research that, for example, considers when evaluations of government will be more and less related to trust. The use of both the ANES and MDS measures in the current research also allows for speculation as to the likely relations among the constructs in situations where they cannot all be measured together.

### Hypothesis three

The third hypothesis—that *Ability*, *Benevolence*, and *Integrity* would mediate the relation between the performance evaluations and trust measured as a willingness to accept vulnerability—was also largely supported in the current data. The CFA results revealed significant bivariate associations for the performance evaluations regarding the economy and how well government has represented the participant’s interests with *W2AV* and the correlate measures. Evaluations of government scandals were also correlated with *W2AV* but were only associated with three of the correlates. When the mediations were added in the structural model, most of the direct effects became non-significant suggesting that they were, in fact, mediated as hypothesized and this was corroborated in significance tests of the indirect effects. Two direct paths (independent of *Trustworthiness*), however, remained significant and to evaluate their importance we assessed the change in model fit associated with setting their regression coefficients to zero, thereby effectively removing these paths from the model. Both changes resulted in statistically significant decreases in model fit but the relative size of this decrement varied somewhat with the larger decrement being associated with removing the effect of evaluations of the economy on *Feelings Thermometer* (-2LLΔ = 44.84). As mentioned above, this is consistent with the notion that this feelings thermometer is something of a different correlate than the others and may better represent a more global trustworthiness-like evaluation.

These results suggest that trustworthiness is likely to be a meaningful mediator of performance evaluations of government. We, therefore, suggest that the process of political trust may be understood as a process in which individuals generate performance evaluations that are then used to make attributions of trustworthiness that then facilitate trust. Thus, in place of a direct effect, the results here suggest a mediated effect such that the reason that performance evaluations lead to trust is their impact on trustworthiness. Note, however, that our results suggest that this mediation is not complete. In particular, *Trustworthiness* struggles tomediate the effect of scandals. Interestingly, the *ANES* measure seems to better mediate this relation, potentially because the measure directly addresses perceptions that government is “crooked.” The analogous component of *Trustworthiness* is *Integrity* but it appears that this was insufficient to account for that relation. Thus, although research often does suggest that ability, benevolence, and integrity are sufficient for accounting for the majority of the variance in trustworthiness evaluations, it may be that government requires an additional component. Potential candidates for addition would include identification and reliability such that scandals may be more strongly associated with the perception that government shares the participants’ own values or that it can be counted on to act predictably, but one especially interesting possibility might be a need to specifically measure a *lack* of integrity.

These analyses also contribute to future work by addressing the possibility of the causal mechanism implied by the model. Although limited in its lack of experimental or longitudinal methods, the current mediation analyses do suggest that the proposed mechanism, whereby performance evaluations impact trust because of their impact on trustworthiness, is reasonable. Additionally, the Hypothesis Three results add more nuance to efforts to position the MDS constructs against the ANES index, specifically by suggesting that the ANES is most functionally similar to the measure of trustworthiness. Thus, although using more precise trustworthiness measures will assist in diagnosing when one or more evaluations are relatively high or low, the measurement of political trust could be advanced by simply adding a three-item measure of a willingness to accept vulnerability to a survey that already included the ANES index. Finally, the current results shed light on opportunities for improvement in work applying the MDS model in the political context. In particular, the Hypotheses Three results suggest that our measure of trustworthiness struggled in its ability to account for the influence of scandals.

## Conclusion

The current research is largely supportive of the potential for this classic organizational model of trust to help in clarifying, supplementing, and expanding existing theories of political trust. The current paper therefore contributes to a growing body of literature in a variety of contexts that speak to the potential for a cross-boundary understanding of trust. Specifically, the theoretical model proposed here joins with work that suggests that trust is best understood as a willingness to accept vulnerability that rests on an assessment of whether the trustee is worthy of that trust. Thus, this model suggests that the success of efforts to understand and build trust likely rely heavily on the extent to which they address salient vulnerabilities but it advances this contribution by suggesting that assessments of ability, benevolence, and integrity have a particularly important role to play.

Our results, although preliminary given their reliance on cross-sectional data from a convenience-based sample, suggest that measures derived from the MDS model are psychometrically sound and that the model-hypothesized relations among the constructs were largely empirically supported. In particular, our results suggest that trustworthiness is a noteworthy mediator of evaluations of the performance of government: Individuals seem to use these performance evaluations to determine the trustworthiness of government and, as a result, the extent to which it should be trusted. Thus, in place of traditional approaches whereby performance evaluations of government were hypothesized to have direct impacts, our research suggests that the reason these evaluations matter may be because they impact perceptions of the trustworthiness of government itself and that these evaluations can be meaningfully captured via assessments of government’s ability, benevolence, and integrity.

The current research therefore provides a first step towards a research agenda that could reinvigorate this area of scholarship with an increasingly sophisticated roadmap of the process by which performance evaluations impact cooperation and compliance with government. In so doing, this work would support greater nuance in efforts to understand and predict trends in political trust. As noted at length, scholars have sought to identify additional factors and mechanisms that explain why traditional approaches to political trust have generally failed to provide predictions that are consistent with data trends. Application of the MDS model suggests that this may be because the mechanisms of the model are inappropriately specified. Thus, researchers may be best served by seeking to better understand the relation between performance evaluations and trustworthiness, instead of assuming a direct relation with trust. Our work suggests that knowing which performance evaluations impact which component(s) of trustworthiness and for whom may be an especially profitable area of research, and there is a significant body of work in political psychology from which specific hypotheses can be drawn. For example, as noted above, research consistently suggests that negative information is perceived as being more diagnostic than is positive information [24]. This would suggest that negative performance evaluations will be more likely to impact trustworthiness than positive evaluations, but yet another area of literature would add additional nuance to this hypothesis. In line with work on political polarization specifically (e.g., [27] and motivated reasoning in general (e.g., [26], there is good reason to believe that performance evaluations that are supportive of the trustor’s party affiliation will also be more likely to impact trustworthiness components [20]. Indeed, it is likely that, in the face of an objectively improving economy, a desire to support one’s party may enhance the connection of improvements in the economy with ability, while a desire to undermine an opposing party may sever this relation. Thus, our work offers an important adjustment to contemporary efforts to understand political trust by shifting the conversation from a discussion of the inconsistent impact of performance evaluations directly on trust defined by its measure, to a more precise and nuanced account that takes into account the potential psychological mechanism of this effect.

## Supporting information

S1 File Randomly assigned condition primes. (DOCX)

S2 File Dataset. Data in CSV format.
(CSV)

S3 File Syntax. Syntax in INP (M*plus*) format. Can be opened as a TXT file.
(INP)

S1 Table Attitudinal items and univariates. (DOCX)
