# Supplementary material for: Understanding the psychological nature and mechanisms of political trust
Source: PLoS One. 2019 May 15;14(5):e0215835. doi: 10.1371/journal.pone.0215835 (PMC6519795; doi:10.1371/journal.pone.0215835)
Supplement: S1 File — (DOCX) [file pone.0215835.s001.docx]

**S1. Randomly Assigned Condition Primes**

The President of the United States

The President of the United States of America (POTUS) is the elected head of state and head of government of the United States. The president leads the executive branch of the federal government and is the commander-in-chief of the United States Armed Forces. Article II of the U.S. Constitution vests the executive power of the United States in the president and charges them with the execution of federal law, alongside the responsibility of appointing federal executive, diplomatic, regulatory and judicial officers, and concluding treaties with foreign powers with the advice and consent of the Senate. The president is further empowered to grant federal pardons and reprieves, and to convene and adjourn either or both houses of Congress under extraordinary circumstances. The President is largely responsible for dictating the legislative agenda of his party and the foreign and domestic policy of the United States.

The president is indirectly elected by the people through the Electoral College to a four-year term, and is one of only two nationally elected federal officers, the other being the Vice President of the United States. The Twenty-second Amendment, adopted in 1951, prohibits anyone from ever being elected to the presidency for a third full term. It also prohibits a person from being elected to the presidency more than once if that person previously had served as president, or acting president, for more than two years of another person's term as president. In all, 43 individuals have served 44 presidencies (Cleveland's two non-consecutive terms each counted) spanning 56 full four-year terms. On January 20, 2009, Barack Obama became the 44th and current president. On November 6, 2012, he was re-elected and is currently serving the 57th term, which ends on January 20, 2017.

The United States Congress

The United States Congress is the bicameral legislature of the federal government of the United States consisting of two houses: the Senate and the House of Representatives. The Congress meets in the Capitol in Washington, D.C. Both senators and representatives are chosen through direct election, though vacancies in the Senate may be filled by a governor's appointment. Members are usually affiliated to the Republican Party or to the Democratic Party, and only rarely to a third-party or as independents. Congress has 535 voting members: 435 Representatives and 100 Senators.

The members of the House of Representatives serve two-year terms representing the people of a single constituency, known as a "district". Congressional districts are apportioned to states by population using the United States Census results, provided that each state has at least one congressional representative. Each state, regardless of population or size, has two senators. Currently, there are 100 senators representing the 50 states. Each senator is elected at-large in his or her state for a six-year term, with terms staggered, so every two years approximately one-third of the Senate is up for election.

The United States Supreme Court

The Supreme Court of the United States (SCOTUS) is the highest federal court of the United States. Established pursuant to Article III of the United States Constitution in 1789, it has ultimate (and largely discretionary) appellate jurisdiction over all federal courts and over state court cases involving issues of federal law, plus original jurisdiction over a small range of cases. In the legal system of the United States, the Supreme Court is the final interpreter of federal constitutional law, although it may only act within the context of a case in which it has jurisdiction.

The Court consists of the Chief Justice of the United States and eight associate justices who are nominated by the President and confirmed by the Senate. There have been 112 justices on the Court since 1790. Once appointed, justices have life tenure unless they resign, retire, take senior status, or are removed after impeachment (though no justice has ever been removed). In modern discourse, the justices are often categorized as having conservative, moderate, or liberal philosophies of law and of judicial interpretation. Each justice has one vote, and while many cases are decided unanimously, many of the highest profile cases often expose ideological beliefs that track with those philosophical or political categories. The Court meets in the United States Supreme Court Building in Washington, D.C.
