# Supplementary material for: Understanding the psychological nature and mechanisms of political trust
Source: PLoS One. 2019 May 15;14(5):e0215835. doi: 10.1371/journal.pone.0215835 (PMC6519795; doi:10.1371/journal.pone.0215835)
Supplement: S1 Table — (DOCX) [file pone.0215835.s004.docx]

**S2.** **Attitudinal Items and Univariates**

| Construct | Item | Mean | SD | Range | Lower Label | Upper Label |
| --- | --- | --- | --- | --- | --- | --- |
| *Eval Economy* | Do you believe that the federal government has hurt or helped the economy? | 2.73 | 1.13 | 1-5 | Mostly hurt | Mostly helped |
| *Eval Interests* | Do you believe that the federal government has represented your interests over the last few years? | 2.40 | 1.21 | 1-5 | Mostly no | Mostly yes |
| *Eval Scandals* | Do you believe that scandals involving the federal government have been a big deal over the last few years? | 2.60 | 1.26 | 1-5 | Mostly no | Mostly yes |
| *ANES Trust* | How much of the time do you think you can trust the government in Washington to do what is right-- just about always, most of the time, or only some of the time? | 36.86† | 20.53 | 0-100 |  |  |
|  | Would you say the federal government is pretty much run by a few big interests looking out for themselves, or that it is run for the benefit of all the people? | 9.59† | 29.48 | 0-100 |  |  |
|  | Do you think that people in the government waste a lot of money we pay in taxes, waste some of it, or don't waste very much of it? | 24.39† | 28.61 | 0-100 |  |  |
|  | Do you think that quite a few of the people running the government are crooked, not very many are, or do you think hardly any of them are crooked? | 19.03† | 27.29 | 0-100 |  |  |
| *Ability* | The federal government is generally competent. | 2.92 | 0.83 | 1-5 | Never | All of the time |
|  | The federal government is capable of performing its job. | 3.08 | 0.84 | 1-5 | Never | All of the time |
|  | The federal government has the knowledge necessary to do the work that needs to be done. | 3.37 | 0.89 | 1-5 | Never | All of the time |
| *Benevolence* | The federal government cares about people like me. | 2.44 | 0.83 | 1-5 | Never | All of the time |
|  | The federal government is concerned about the welfare of people in situations like mine. | 2.49 | 0.83 | 1-5 | Never | All of the time |
|  | The federal government looks out for what is important to people similar to me. | 2.49 | 0.82 | 1-5 | Never | All of the time |
| *Integrity* | The federal government sticks to its word. | 2.64 | 0.80 | 1-5 | Never | All of the time |
|  | The federal government adheres to a strong moral code. | 2.40 | 0.88 | 1-5 | Never | All of the time |
|  | The words and actions of the federal government are consistent. | 2.60 | 0.85 | 1-5 | Never | All of the time |
| *W2AV Trust* | I am open to letting the federal government make more decisions about issues that are important to me. | 2.44 | 0.92 | 1-5 | Never | All of the time |
|  | I am comfortable with the federal government’s control over my future. | 2.27 | 0.92 | 1-5 | Never | All of the time |
|  | I am willing to let the federal government resolve problems that are critical to me, even though I cannot monitor all of its actions. | 2.58 | 0.97 | 1-5 | Never | All of the time |
| *Tax Item* | Given that federal income taxes are the primary source of funding for much of the government, do you feel you are asked to pay more than you should, about the right amount, or less than you should? | 2.26 | 0.88 | 1-5 | I am asked to pay much more than I should | I am asked to pay a lot less than I should |
| *Feelings Therm.* | On a scale of 1 to 100, where 1 is cold and 100 is warm, how do you feel about the federal government in Washington? | 37.53 | 22.04 | 0-100 | Cold | Warm |
| *Cross-Party Cooperation* | I would encourage members of my political party to try harder to work with other political groups in Washington, even if that means compromise. | 5.05 | 1.43 | 1-7 | Strongly disagree | Strongly agree |
| *Monitoring* | I would support government monitoring of American's financial or social media accounts without a warrant to watch for suspicious activity that might be connected to terrorism. | 2.89 | 1.88 | 1-7 | Strongly disagree | Strongly agree |
| *Evacuation* | If the federal government ordered an evacuation in my area ahead of a major disaster (e.g., a hurricane), I would comply. | 5.78 | 1.17 | 1-7 | Strongly disagree | Strongly agree |
| *Vaccination* | If the federal government recommended a new vaccination, I would get it. | 4.40 | 1.67 | 1-7 | Strongly disagree | Strongly agree |

† denotes recoded values.
